# Muguet

**Published:** 1852-09

**Authors:** 


*Muguet.*

From *Malgaigne's Medico Chirurgical Review* we translate the following notice of an affection called Muguet, a species of vegetation of the mucous membrane. M. Gubler, after having made some observations on the nature of this affection, says:

"Having been called to make post-mortem examinations of several young subjects who had died while they were affected with Muguet, I have been able to make some investigatious in regard to the precise seat of these productions. Without entering into the detail of particular observations, I think I may put forth the following propositions:—

"*First.*—The affection known by the name of Muguet commences by a certain inflammation of the superior part of the alimentary tube.

"*Second.*—This inflammation seems to cause a suppression of the salivary secretion, which is alkaline, and perhaps an exaggeration of the acidity proper to the bucal mucus, which continues to be secreted, and manifests an energetic re-action on the paper of tournesol (sunflower).

"*Third.*—In the presence of this acid condition of the mouth, seconded by a temperature sufficiently elevated, cryptogamic vegetations are not slow in developing themselves on the dorsal surface of the tongue, the palate, the vail of the soft palate and the pharynx itself, upon the internal portion of the check which is comprised between the dental arches when the jaws are separated, and upon those parts of the lips which come in contact with the gums or teeth.

"*Fourth.*—It is to be remarked that the parts of the mouth ordinarily preserved are only those which are not directly accessible to the atmospheric air. The influence of this agent in producing those vegetations, *mucedinces* is so real that I remember only one instance of their being found in the œsophagus, and never in the stomach, where otherwise they would be able to subsist, except in the absence of the gastric juice.

"*Fifth.*—These *mucedinces* have their origin in the interior of the glands which open upon the surface of the tongue, the lips and other parts of the mouth, as well as in the saburral coating which covers the first of these organs. The opithelial cells and the particles of coagulated casein which constitute this coating as well as the altered mucous of the glands constitute a sort of *humus* well adapted for the development of these false parasites.

"*Sixth.*—These filaments having their roots in the glandular cavity, by increasing in length and number they fill up at length cavity, and, escaping from it, continue to spread over the neck of the follicle and meet on all sides, forming a small rounded eminence of milky whiteness! The appearance of the production recalls sufficiently well the form of a grenade.

"*Seventh.*—If the orifice is too small, the linen-like filaments distend the glands beyond measure, and thin their walls till they seem to form sub-epithelial tumors. I have never clearly seen grains of muguet situated between the elevated epithelium and the surface of the mucous erm, although I by no means deny the possibility of this variety of form.

"*Eighth.*—It appears from the preceding that the *mucedinees* of muguet do not attack the living tissues, but that they develope themselves simply in the midst of organic detrius in determinate conditions, and that their appearance is only an epi-phenomenon in the disease."

From the foregoing, it seems that alkaline remedies are indicated to destroy the parasites, and, in addition, medicines to correct the condition of the secretions upon which their existence depends.

